# Universalização do abastecimento de água em áreas rurais: a entrada na agenda de Marechal Cândido Rondon, Paraná, Brasil

**DOI:** 10.1590/0102-311XPT036124

**Published:** 2025-03-31

**Authors:** Vitor Prá de Athayde, Sonaly Rezende

**Affiliations:** 1 Universidade Federal de Minas Gerais, Belo Horizonte, Brasil.

**Keywords:** Saneamento Básico Rural, Abastecimento de Água, Participação Comunitária, Políticas Públicas, Rural Basic Sanitation, Water Supply, Community Participation, Public Policies, Saneamiento Básico Rural, Abastecimiento de Agua, Participación Comunitaria, Políticas Públicas

## Abstract

O Município de Marechal Cândido Rondon, Paraná, Brasil, declara que toda a sua população é atendida pelo serviço de abastecimento de água, inclusive no meio rural, destacando-se frente ao cenário de déficit de atendimento observado no Brasil. Este trabalho se propõe a estudar esse caso de sucesso, buscando interpretar como o abastecimento de água universal em áreas rurais entrou na agenda do município para se efetivar em uma política pública exitosa. A fundamentação teórica utilizada foi o Modelo de Fluxos Múltiplos, apresentado por Kingdon. A coleta de dados foi realizada por meio de análise documental e de entrevistas semiestruturadas com informantes-chave. O material obtido foi submetido a uma análise de conteúdo qualitativa, que indicou a participação de três grupos de agentes como primordial para alinhamento dos fluxos decisórios. A atuação dos moradores contribuiu para a evidenciação dos problemas relacionados à ausência do abastecimento de água adequado. Membros do Serviço Autônomo de Água e Esgoto (SAAE), autarquia municipal, atuaram no desenvolvimento e na propagação de uma solução considerada viável, caracterizada pela gestão comunitária e pelo uso de tecnologias de operação simplificada. Por fim, anos após a concepção dos primeiros projetos, o Poder Executivo municipal, tornou o abastecimento de água universal uma prioridade no meio político, proporcionando a ascensão do tema à agenda. Constatou-se, então, que definir a universalização como um objetivo prioritário foi fundamental para sua obtenção, o que subsidia a atuação de agentes que buscam ascender a universalização à agenda em outras localidades.

## Abstract (en)

The Marechal Cândido Rondon municipality, Paraná State, Brazil, declares that its entire population is served by its water supply service, including those in rural areas, standing out in the Brazilian service deficit scenario. This study proposes to evaluate this success story to interpret how the universal water supply in rural areas entered the agenda of the municipality toward an effective and successful public policy. The Multiple Streams Framework by Kingdon was used as the theoretical foundation. Data were collected by document analysis and semi-structured interviews with key informants. The obtained material was subjected to a qualitative content analysis that indicated the participation of three groups of agents as essential for aligning decision-making flows. Residents’ participation helped to highlight the problems related to the absence of adequate water supply. Members of the Autonomous Water and Sewage Service (SAAE, acronym in Portuguese), a municipal autarchy, developed and disseminated a viable solution characterized by community management and simplified operation technologies. Finally, years after the conception of the first projects, the municipal executive branch prioritized universal water supply in the political environment, providing the inclusion of the theme to the agenda. Defining universalization as a priority objective was fundamental for its achievement, subsidizing the performance of agents who seek to raise universalization to the agenda in other locations.

## Abstract (es)

El municipio de Marechal Cândido Rondon, Paraná, Brasil, informa que provee el abastecimiento de agua a toda su población, incluida la de zonas rurales, lo que se destaca frente al déficit de servicio en Brasil. Este trabajo tiene como objetivo analizar este caso de éxito para identificar cómo el abastecimiento universal de agua en zonas rurales se incluyó en la agenda de este municipio y logró ser una política pública exitosa. El marco teórico utilizado fue el Modelo de Corrientes Múltiples, presentado por Kingdon. La recopilación de datos se realizó mediante análisis documental y entrevistas semiestructuradas con informantes clave. El material obtenido fue sometido a un análisis de contenido cualitativo, que indicó la participación fundamental de tres grupos de agentes para alinear los flujos de la toma de decisiones. El desempeño de los residentes contribuyó a resaltar los problemas relacionados con la falta de suministro adecuado de agua. Los miembros del Servicio Autónomo de Agua y Alcantarillado (SAAE, por su sigla en portugués), entidad municipal, trabajaron en el desarrollo y propagación de una solución considerada viable, caracterizada por la gestión comunitaria y el uso de tecnologías de operación simplificadas. Tras años de la concepción de los primeros proyectos, el Poder Ejecutivo municipal convirtió el abastecimiento universal de agua en una prioridad en el entorno político, incluyendo el tema en la agenda. Por lo tanto, se constató que definir la universalización como un objetivo prioritario fue fundamental para su consecución, lo que subvenciona el desempeño de los agentes que buscan que la universalización de este acceso forme parte en la agenda en otras localidades.

## Introdução

A relação entre serviços de saneamento e saúde é recorrente nas discussões técnico-científicas, e sua adequada compreensão pode contribuir para o alcance da universalização do acesso ^1^. A prevenção de doenças por meio do saneamento é um aspecto fundamental nessa interação, tendo sido abordada em profundidade na literatura ^2^. Ao se considerar a perspectiva da promoção da saúde, a importância do saneamento se expande, abrangendo também uma dimensão sociocultural e política ^3^. Assumir essa perspectiva garante, então, que o acesso universal ao saneamento seja visto como essencial em qualquer contexto, não apenas em situações em que há o risco de doenças, já que a prestação adequada e a perenidade desses serviços promove o bem-estar do indivíduo ^4^.

Esse entendimento, além de propagado por especialistas da área, foi incorporado na legislação nacional por meio da *Lei nº 11.445*, de janeiro de 2007, que estabelece as diretrizes nacionais para o saneamento básico e traz a universalização e a articulação com as políticas de promoção da saúde como dois dos princípios fundamentais da prestação dos serviços públicos de saneamento ^5^. Apesar disso, o déficit de atendimento ainda perdura no Brasil, assim como nos países em desenvolvimento, em alguns estratos da população que se encontram fora do escopo de prioridades do poder público ^6^ ^,^ ^7^ ^,^ ^8^, em áreas rurais e em assentamentos precários urbanos. O déficit persistente também afeta desproporcionalmente as mulheres e as crianças, por vezes impondo severas limitações aos processos educacionais e de formação profissional, além de afetarem sobremaneira a saúde desses grupos ^9^.

Considerando que a tomada de decisão e o dinamismo histórico-temporal são inerentes às políticas públicas ^10^, entende-se que, no contexto brasileiro, o reconhecimento legal dos princípios que guiam o saneamento não foi suficiente para motivar - ou possibilitar - a atuação efetiva dos agentes políticos em prol da universalização. No Brasil, a agenda política contemporânea do saneamento segue priorizando ações de natureza estrutural ^11^ em áreas urbanas das regiões mais dinâmicas do ponto de vista econômico, e os modelos de gestão de saneamento têm demonstrado diferenças na prestação de serviços entre os estratos socioeconômicos mais desfavorecidos, sobretudo em áreas rurais ^12^ ^,^ ^13^. A decisão desses agentes em atuar num certo tema não é determinada pela relevância da questão, mas sim pela interpretação social e política que lhe é atribuída ^14^, processo sujeito à influência de condicionantes políticos, econômicos e culturais ^10^.

No âmbito do estudo das políticas públicas, portanto, é necessário entender, em cada situação, o que faz uma questão ascender à agenda e ser objeto das ações públicas ^15^. Este trabalho buscará aplicar essa perspectiva para análise de um caso exitoso quanto à universalização no âmbito do saneamento do Município de Marechal Cândido Rondon. Situado no oeste do Estado do Paraná, Brasil, o município declara que toda a sua população, de 55.836 habitantes em 2022 ^16^, é atendida pelo serviço de abastecimento de água ^17^. Justifica-se, então, a escolha de Marechal Cândido Rondon como objeto de estudo, principalmente ao se considerar que sua ocupação territorial não se restringe ao meio urbano. Com mais de 16% de seus habitantes residindo em áreas rurais no ano de 2010 ^18^, constata-se que o déficit no atendimento foi superado também onde ele tem se mostrado mais persistente no Brasil, o meio rural, e por isso esse foi o foco desta pesquisa.

Nessas localidades da cidade é adotado um modelo descentralizado, em que associações comunitárias são encarregadas da gestão e operação dos sistemas de abastecimento de água. Esses sistemas são majoritariamente compostos pela captação de água bruta por poço tubular profundo e pelo tratamento simplificado da água por cloração ^19^. Os próprios moradores, membros das associações, operam os cloradores, efetuam reparos na rede e realizam a cobrança dos demais usuários ^20^. Ao Serviço Autônomo de Água e Esgoto (SAAE), autarquia municipal de Marechal Cândido Rondon, coube a implantação dos sistemas e o apoio às associações ^17^ por meio, por exemplo, do monitoramento da qualidade da água, do assessoramento administrativo e do auxílio técnico em reparos de maior complexidade ^21^.

A gestão do abastecimento de água em Marechal Cândido Rondon já foi objeto de outros estudos ^19^ ^,^ ^20^ ^,^ ^21^ ^,^ ^22^, que discutem principalmente a forma como é realizado o atendimento às comunidades rurais e algumas de suas implicações. Apesar de apresentarem elementos descritivos relacionados à criação dos sistemas rurais de abastecimento, nenhum deles se debruçou sobre as condicionantes que provocaram a busca pela universalização do atendimento, carecendo de interpretações que explicam esse processo e evidenciem as motivações dos agentes envolvidos. Assim, o objetivo deste estudo é responder a essas questões, elucidando como o abastecimento de água universal em áreas rurais entrou nas agendas governamental e decisória de Marechal Cândido Rondon.

## Metodologia

### Fundamentação teórica

A fundamentação teórica adotada neste trabalho foi o Modelo de Fluxos Múltiplos desenvolvido por Kingdon em 2003 ^23^, que apresenta uma explicação abrangente quanto ao processo de mudanças políticas ^24^. Por estabelecer um enfoque na dinâmica das ideias que interferem no desenvolvimento de políticas públicas e formam a agenda ^25^, o modelo possui um nítido alinhamento com as premissas aqui discutidas. Além disso, sua aplicação neste estudo também é amparada pela literatura do setor, já que o modelo tem sido amplamente utilizado no estudo de políticas públicas ^26^, inclusive em pesquisas relacionadas ao saneamento no Brasil ^27^ ^,^ ^28^ ^,^ ^29^.

Em seu trabalho, Kingdon discute a formação da agenda governamental, composta pelo conjunto de assuntos que ganham atenção dos atores ligados ao governo, e da agenda decisional, um subconjunto de assuntos da primeira, restrita aos temas em que os formuladores de políticas decidem efetivamente atuar. De acordo com o Modelo de Fluxos Múltiplos, a abertura de uma janela de oportunidade permite a convergência de três fluxos decisórios - o de problemas, o de soluções e o da política -, e esse acoplamento de fluxos é o que provoca mudanças na agenda ^23^.

O primeiro fluxo se refere ao processo de reconhecimento de situações como problemas. Como mencionado, essa percepção é formada por uma construção social e política relacionada à interpretação e influência de diferentes agentes, que passam a reconhecer uma questão como digna de atenção e passível da atuação das políticas públicas. O segundo fluxo compreende a geração e a propagação de soluções, que surgem em um processo dinâmico. Especialistas em determinadas áreas criam, adaptam e combinam ideias que, a depender da percepção de sua viabilidade, podem prosperar como alternativas para solucionar problemas. Por fim, o terceiro fluxo, que se mostra um importante formador de agenda, compreende a esfera política. Nele, é percebida uma dinâmica própria em que processos de barganha e negociação definem coalizões e interferem nas decisões dos formuladores de políticas ^23^.

**Figura 1:**
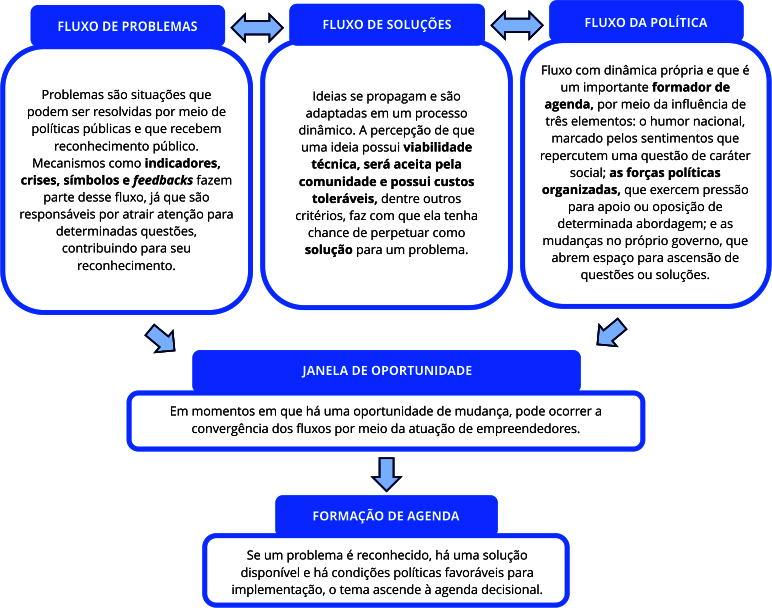
Representação esquemática dos fluxos decisórios segundo o Modelo de Fluxos Múltiplos.

Quando os três fluxos convergem, ou seja, o problema é reconhecido, uma solução está disponível e há condições políticas favoráveis à sua implementação, surge uma oportunidade para que uma questão ascenda a agenda decisional e se concretize em uma política pública ^25^, conforme representado na Figura 1. Esses conceitos guiaram as discussões desenvolvidas nesse trabalho, sendo que a principal adaptação necessária para aplicação do modelo foi a alteração do escopo de análise: da agenda nacional, como apresentado por Kingdon, para a agenda municipal.

### Coleta e análise de dados

A coleta de dados teve como ponto de partida a análise documental, após consulta de publicações oficiais, do município e do SAAE, e científicas, relacionadas ao caso em estudo. O conhecimento prévio obtido a partir desse material foi utilizado para elaboração de roteiros de entrevistas semiestruturadas ^30^ voltadas ao tema, as quais formaram a etapa principal de coleta de dados do trabalho. Durante um período de dez dias, foram realizadas 15 entrevistas com informantes-chave, representantes de dois grupos: membros do SAAE, instituição que concentrou a atuação do Município no desenvolvimento dos projetos, e membros das comunidades, que realizam a gestão dos sistemas rurais de abastecimento de água.

Foram escolhidos cinco entrevistados do SAAE por meio de uma seleção primária ^30^, buscando-se informantes envolvidos no processo de universalização, lotados em diferentes setores da autarquia (administrativo, operacional, financeiro, engenharia e assessoria de águas rurais). Dentre os quarenta sistemas rurais em operação em Marechal Cândido Rondon, adotou-se uma amostragem intencional para realização de entrevistas com os moradores. Foram seguidos os pressupostos da maior variabilidade, da seleção de casos críticos e da conveniência, buscando-se maximizar o número de comunidades visitadas no tempo disponível e abranger localidades com características diversas. O contato com os moradores foi intermediado pelo SAAE, que indicou membros das associações que participam da administração e da operação dos sistemas, totalizando dez entrevistados. Foram atribuídos códigos como forma de garantir o anonimato dos participantes, sendo de ES1 a ES5 os membros do SAAE e de EA1 a EA10 os membros das associações.

O conteúdo das entrevistas foi transcrito e submetido a uma análise de conteúdo qualitativa, seguindo as diretrizes de Schreier ^31^. Cada entrevista consistiu em uma unidade de análise, que foi submetida a uma segmentação temática ^31^ com exclusividade mútua de códigos. A estrutura de codificação adotada, demonstrada no Quadro 1, foi criada a partir da combinação das estratégias de orientação por conceito e de orientação pelos dados, em que o referencial teórico definido por Kingdon e os elementos identificados em uma análise prévia do material determinaram as categorias de análise.

**Quadro 1:** Estrutura de codificação utilizada para análise de conteúdo qualitativa.

DIMENSÃO DE ANÁLISE	CÓDIGOS	
1. Entrada na agenda (análise do processo de formação da agenda, segundo os fluxos múltiplos de Kingdon)	Fluxo dos problemas	Disponibilidade de água
		Qualidade de água
	Fluxo das soluções	Geração de alternativa
		Difusão de alternativa
	Fluxo da política	Mudanças institucionais
		Projeto de governo
		Demanda e humor popular
		Apoio extramunicipal
2. Contexto (identificação da conjuntura em que se estabeleceu o processo de entrada na agenda)	Contexto político	
	Contexto econômico	
	Contexto social	
	Contexto dos serviços de saneamento	

A codificação foi realizada por um único pesquisador e, por isso, foi submetida a um teste. Sendo realizada uma segunda codificação de 20% do material 14 dias após a primeira, obteve-se uma correspondência entre elas de 91% para a dimensão de análise 1 e 93% para a dimensão de análise 2. A estabilidade dos resultados atestou a consistência dos códigos definidos, que então foram utilizados para classificação de todo o material. A descrição e a interpretação dos resultados obtidos por essa análise de conteúdo qualitativa são apresentadas nos tópicos a seguir. Destaca-se que este projeto foi aprovado pelo Comitê de Ética em Pesquisa da Universidade Federal de Minas Gerais (CAAE 71663923.8.0000.5149).

## Resultados e discussão

### Fluxo de problemas

O SAAE de Marechal Cândido Rondon foi criado em 1966 ^32^, mas até o fim da década de 1980, sua atuação na prestação do serviço de abastecimento de água foi restrita ao distrito sede e aos distritos rurais, representados por aglomerações de domicílios. Nas áreas rurais de ocupação dispersa, conforme relatado pelos moradores, predominava a utilização, em cada propriedade, de poços rasos para captação de água. Não havendo participação do poder público na implantação, as soluções apresentavam fragilidades, tanto em relação à qualidade da água quanto em relação à disponibilidade. Mesmo que essas condições gerem efeitos prejudiciais à saúde da população, a percepção de que representam um problema está ligada a elementos interpretativos (como discutido) e depende de mecanismos que chamem atenção para a questão.

Quanto à qualidade da água, a percepção do problema no município ocorreu devido aos mecanismos definidos por Kingdon como “indicadores”. Segundo os relatos de ES2 e ES3, no início da década de 1980, estudos realizados em decorrência da instalação da Usina Hidrelétrica de Itaipu na região indicaram a presença de pesticidas na água consumida no meio rural. Os resultados evidenciaram que a pequena profundidade dos poços utilizados os tornava vulneráveis à contaminação, o que gerava riscos à saúde, conforme relatado por ES2: “*A gente sabia que todo o interior, todos os poços estavam com contaminação. Ao ponto de que isso foi uma das grandes questões da saúde que foram invocadas e da necessidade de o poder público começar a agir*”. Então, denota-se que, sob a ótica da saúde, o início da atuação municipal no saneamento rural foi motivado pelo caráter preventivo de doenças.

Em relação à disponibilidade de água, todos os moradores entrevistados relataram a existência de dificuldades, seja em suas próprias comunidades ou em localidades próximas. A evidenciação desse problema se deu principalmente pelo grupo de mecanismos definidos por Kingdon como “eventos, crises e símbolos”. Segundo o Método de Fluxos Múltplos, com a repercussão de situações extremas, esses elementos atraem a atenção dos tomadores de decisão para uma determinada questão ^23^.

No caso em estudo, a limitação do acesso à água em algumas localidades foi evidenciada (e agravada) em períodos de seca, quando a baixa reposição de águas subterrâneas reduzia, ou até interrompia, a vazão dos poços rasos. Segundo EA6, o poço instalado em sua propriedade apresentava vazão de apenas 100 litros de água por dia nos períodos de pouca chuva, sendo insuficiente para atender as necessidades de sua família. A partir da reivindicação dos moradores frente a representantes do poder público, esses cenários de crise atraíram atenção para o problema. A criação do projeto de abastecimento rural foi então motivada pela baixa disponibilidade de água em algumas localidades, como relatado por ES1 e ES2.

Dessa maneira, os relatos demonstraram que a atuação dos formuladores de políticas foi motivada pela percepção de problemas, mas corroboram a ideia de que, sozinho, esse reconhecimento não é suficiente para concretização de ações, como apresentado por Kingdon. No início da década de 1980, os problemas de indisponibilidade e má qualidade da água já eram reconhecidos, mas a instalação do projeto de abastecimento rural só foi iniciada em 1989. Portanto, apesar de já permear a agenda governamental devido ao fluxo dos problemas, o abastecimento de água rural só pode ascender à agenda decisional com a convergência de outros fatores pertencentes aos demais fluxos, como discutido a seguir.

### Fluxo de soluções

O Método de Fluxos Múltiplos descreve que, no fluxo de soluções, a escolha de alternativas a serem implementadas ocorre em um processo dinâmico de criação e adaptação de ideias ^23^, o que foi observado no caso em estudo. Antes da implantação do modelo de gestão comunitária em Marechal Cândido Rondon, esse tipo de solução já era propagado por especialistas do setor de saneamento em outras localidades. As ações de saneamento rural financiadas pelo Banco Mundial, no decorrer da década de 1980, por exemplo, se caracterizavam, dentre outros aspectos, pela operação dos sistemas pelas associações comunitárias e adoção de tecnologias de baixo custo e fácil operação ^33^, elementos que se fizeram presentes em Marechal Cândido Rondon. No Brasil, a gestão perene e qualificada dos sistemas rurais por associações comunitárias também já era defendida por especialistas, estando presente no Plano Nacional de Saneamento Rural (PNSR) desenvolvido entre 1985 e 1989 ^27^.

Os entrevistados indicaram que, em Marechal Cândido Rondon, a geração de alternativas se deu, principalmente, pela atuação do SAAE, que contou, inicialmente, com apoio da Companhia de Saneamento do Paraná (Sanepar). Segundo ES4, o primeiro projeto de abastecimento de água na zona rural “*foi elaborado via convênio com a Sanepar, Governo do Estado. Então, veio o técnico da Sanepar, fez um projeto, levantamento pra nós. Daí o SAAE depois executou* (...) *em consórcio com os agricultores*”. A partir desse projeto piloto, as ideias pré-existentes foram sendo adaptadas à realidade do município, pelos próprios membros do SAAE. De acordo com ES3, a concepção do projeto ocorreu de “... *forma simples. Para nós, uma forma meio que automática, muito simples, isso foi se criando*”, o que enaltece o dinamismo associado por Kingdon ao fluxo das soluções.

Segundo o Modelo de Fluxos Múltiplos, o atendimento a critérios de seleção aumenta a chance de uma alternativa prevalecer como solução para um problema ^23^. Nesse sentido, os relatos indicam que o modelo de gestão comunitário era aderente ao senso de associativismo já existente na cultura da população rural do município, como apresentado por ES3: “*Em alguns lugares já existiam as associações comunitárias e esse pessoal participava muito*”. Esse elemento também aumentava a viabilidade do modelo, já que a participação social era um fator essencial para seu sucesso. Além disso, a formação de associações foi motivada pela percepção da inviabilidade de soluções individuais, segundo ES2, e “*para diminuir o custo do povo*”, segundo EA9.

No que se refere à escolha da técnica empregada, também foi observado o atendimento aos critérios de seleção. A adoção de captação subterrânea foi tida como viável devido às características da água disponível, que permitiam que o tratamento fosse de baixo custo e complexidade. Além disso, esse tipo de captação já era empregada no município, sendo esperada a aceitação dos usuários à tecnologia frente a uma possível recusa a outras soluções. Essa recusa foi evidenciada em alguns relatos, como no de EA1, que admitiu ter receio em consumir água captada em um rio, mesmo que tratada pelo SAAE.

Dessa maneira, uma solução para o problema já reconhecido de abastecimento de água em comunidades rurais foi estabelecida em Marechal Cândido Rondon. Concebida, inicialmente, na forma de um projeto piloto, a solução criada passou a ser implementada em algumas comunidades, indicando que a prestação desses serviços em áreas rurais já fazia parte da agenda do município.

Kingdon descreve o processo de propagação de soluções, definido como *soften up*, como aquele em que empreendedores buscam promover a aceitação entre formuladores de políticas e o público em geral de alternativas tidas como viáveis ^23^. Em Marechal Cândido Rondon, essa difusão foi realizada principalmente pelos membros do SAAE, como descrito por ES1: “*A partir do momento do interesse dessa comunidade a gente convocou todos* [os potenciais usuários] *para uma reunião.* (...) *Lá se apresentou, então, como funcionaria o sistema, a parte que caberia a eles pagarem, a criação da associação que gerenciaria esse sistema e a apresentação de um estatuto que eles deveriam seguir.* (...) *Discutiu-se e aprovou-se isso em assembleia*”. Mesmo existindo uma resistência inicial de alguns moradores, ES2 e ES3 relatam que o diálogo direto com os usuários, apresentando detalhadamente o projeto e convencendo-os dos benefícios gerados a si e aos demais, possibilitou o sucesso na criação dos sistemas.

### Fluxo da política

No âmbito político, um dos elementos que exercem influência sobre a agenda é o “humor nacional”, caracterizado pelo compartilhamento de uma visão comum acerca de um tema ^23^. Em Marechal Cândido Rondon, constatou-se que o humor popular era favorável ao projeto de implantação dos sistemas rurais de abastecimento, o que incentivou a atuação dos agentes políticos, conforme descrito pelo Modelo de Fluxos Múltiplos. A resolução do problema do abastecimento de água inadequado era um interesse dos moradores, e, mais do que isso, também uma demanda consolidada, o que motivou o poder público a agir. Segundo ES1, o início do projeto se deu “*pela grande procura dos agricultores, contra a falta de água*”, afirmando, devido à importância do setor para o município, que “*se não tem água pra sustentar o agronegócio, o nosso sistema, ele também acaba enfraquecido*”.

Esse trecho também se relaciona a outro elemento do espectro político indicado por Kingdon como formador de agenda: o das forças políticas organizadas. Frequentemente reunidos em cooperativas ou associações, os agricultores exerciam pressão política para expansão do serviço de abastecimento de água nas localidades em que habitavam. Além desses, os membros do SAAE constituíam outro grupo de interesse favorável ao projeto, já que, de acordo com os relatos, consideravam a cessão dos sistemas rurais às comunidades como alternativa necessária para viabilizar o atendimento. Então, os dois principais grupos interessados no projeto eram consensuais quanto ao benefício de sua implantação. Além disso, os entrevistados não indicaram a existência de forças políticas organizadas contrárias à solução proposta. Segundo os relatos, a mobilização política das comunidades era favorável, não emergindo conflitos na implantação da política. Segundo ES2, “*As pessoas viam isso como uma necessidade e todo mundo participava, independente de questão partidária.* (...) *Todo mundo se engajava para o bem da saúde da pessoa*”.

**Figura 2:**
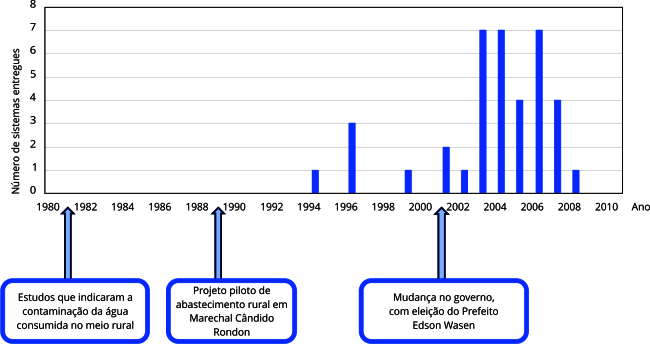
Linha do tempo da entrega dos sistemas rurais de abastecimento de água geridos por associações comunitárias em Marechal Cândido Rondon, Paraná, Brasil.

Com o apoio popular e dos grupos interessados, os sistemas de abastecimento de água passaram a ser implantados, porém em ritmo lento. Segundo ES4, havia descontinuidade de investimentos e obras eram interrompidas antes de sua conclusão. Assim, da concepção do projeto piloto, em 1989, até o ano de 2000, cinco dos sistemas que hoje operam com associações em Marechal Cândido Rondon haviam sido concluídos e entregues à gestão das comunidades ^34^. A partir de 2001, porém, houve uma mudança no ritmo de implantação, com outros 34 sistemas sendo entregues até o ano de 2008, conforme apresentado na Figura 2. De acordo com os relatos, essa aceleração ocorreu a partir de uma mudança no governo municipal, sendo o terceiro elemento político descrito no Modelo de Fluxos Múltiplos que interferiu na formação da agenda. Destaca-se que o Prefeito de Marechal Cândido Rondon detém forte influência sobre as práticas de saneamento no município. Diferentemente do que ocorre em outros modelos de gestão, como na prestação por empresas privadas ou por companhias estaduais, o modelo empregado em Marechal Cândido Rondon atribui ao prefeito o poder de nomear e exonerar os diretores do SAAE, o que o permite direcionar a atuação da autarquia.

A eleição do Prefeito Edson Wasen (Partido da Frente Liberal - PFL) em 2001, após oito anos de governo do Partido do Movimento Democrático Brasileiro (PMDB) representou uma alternância de poder entre os dois principais grupos políticos que governavam Marechal Cândido Rondon até o início dos anos 2000 ^35^. Esse momento de mudança foi apontado pelos entrevistados como crucial para alteração de agenda. Segundo ES4, “*quando entrou um prefeito novo, ele falou: ‘eu vou investir nas águas rurais, eu vou fazer 100% das redes’*”, e a definição desse objetivo determinou o avanço acelerado da implantação dos sistemas. Com um projeto de governo voltado à universalização do abastecimento de água, essa questão passou a fazer parte da agenda de Marechal Cândido Rondon.

Ressalva-se que, apesar dos relatos indicarem a ausência de conflitos políticos relacionados ao tema, foram apresentados por alguns entrevistados questionamentos quanto às motivações dos agentes municipais envolvidos. EA7, por exemplo, indica que a construção da rede de água em sua comunidade, que ocorreu em um ano eleitoral, seria usada para promoção política, já que nesse período, “*cada um quer fritar um pouco a cebola dele*”. De maneira similar, ES4 relata que, por interesses políticos próprios, o prefeito definia comunidades a serem priorizadas: “*Muitas vezes era coisa política, o prefeito indicava: ‘Não, nós vamos fazer essa agora’*”.

Nesse contexto, com o interesse do Executivo municipal, constatou-se que o fluxo político foi responsável pela abertura de uma janela de oportunidade para convergência quanto ao tema da universalização. Entretanto, apesar do destaque atribuído pelos entrevistados à eleição do novo prefeito, ressalta-se que as forças de mudança de agenda são estruturais ^23^, sendo necessário entender a relação entre os três fluxos que oportunizou a ascensão do tema à agenda decisional, como será discutido a seguir.

### A janela de oportunidade e a convergência de fluxos

A dinâmica dos fluxos decisórios, detalhada nos itens anteriores com base nas entrevistas realizadas, indicou que a entrada do abastecimento de água em áreas rurais na agenda governamental de Marechal Cândido Rondon foi provocada, inicialmente, pelo fluxo de problemas. A partir da ocorrência de crises hídricas, que geravam reivindicações dos moradores, e da apresentação de indicadores de contaminação da água, resultantes de estudos realizados na década de 1980, a ausência de abastecimento de água adequado passou a ser percebida como um problema e recebeu atenção do governo municipal.

Apesar de sua presença na agenda, ações relacionadas ao tema só passaram a ser efetivadas quando houve a percepção de que existia uma solução disponível. Esse fenômeno é previsto pelo Modelo de Fluxos Múltiplos, que indica que, sozinho, o fluxo de problemas, assim como o da política, pode ascender um tema à agenda governamental, enquanto o fluxo de soluções, ao se acoplar aos demais, é capaz de promover esse tema da agenda governamental para a decisional. O acoplamento entre o fluxo de problemas e de soluções ocorreu, então, quando os especialistas passaram a perceber uma alternativa disponível como viável, a partir da concepção do projeto piloto de implantação dos sistemas rurais, em 1989. Na visão desses agentes, a solução proposta, com gestão comunitária e captação majoritariamente subterrânea, apresentava viabilidade, custos toleráveis e elevado potencial de aceitação, o que motivou o início da atuação do poder público.

Há de se ressaltar que o abastecimento de água em áreas rurais e a universalização da prestação desse serviço são duas questões diferentes quando se trata da análise da entrada na agenda decisional. É possível (e frequente) que uma demanda ganhe espaço na agenda sem que ela abranja toda a população, estando voltada a determinados grupos. O atendimento universal é um caso singular de prestação de serviços públicos, configurando-se como uma pauta por si só. O desenvolvimento do projeto em Marechal Cândido Rondon demonstrou que, mesmo a universalização sendo resultado da expansão progressiva dos serviços, a entrada do tema do abastecimento de água na agenda não era suficiente para que os formuladores de políticas buscassem atender toda à população. Conforme relatado, até o ano de 2000, o ritmo de expansão era lento e os investimentos no setor eram esporádicos. A escolha das localidades que receberiam os sistemas de abastecimento ocorria a partir da reivindicação dos moradores, sendo, então, restrita aos locais em que houvesse a percepção da indisponibilidade de água de qualidade como um problema a ser resolvido, não abrangendo toda a população.

O Modelo de Fluxos Múltiplos denomina como *spillover* um processo em que, através do estabelecimento de novos princípios, a entrada de um tema na agenda contribui para que temas relacionados também recebam atenção. Tal fenômeno foi constatado em relação à entrada do abastecimento de água rural na agenda de Marechal Cândido Rondon, que, apesar de não ter sido suficiente para obtenção da universalização, contribuiu para que o atendimento universal ascendesse à agenda. A atuação do poder público na implantação dos primeiros sistemas criou um novo precedente, pois até então, a captação de água era uma atribuição exclusiva dos moradores. Ao apresentar resultados positivos, esse novo paradigma despertou interesse das demais comunidades e contribuiu para a entrada do tema na agenda, como relatado por ES3: “*Quando eles viram que o sistema começou a funcionar, daí as comunidades vinham atrás: ‘Ah, nós também queremos esse sistema’* (...) *E os próprios vereadores acabaram falando: ‘vamos ligar* [a rede de água]*, vamos participar da comunidade’. É uma oportunidade para eles também*”.

Com os fluxos dos problemas e das soluções já alinhados, como descrito anteriormente, a abertura da janela de oportunidade se deu a partir do fluxo da política. Em um momento de troca de governo, propício para mudanças de agenda, o Prefeito Edson Wasen definiu, em 2001, a universalização do abastecimento de água como projeto de governo. Contando com apoio popular e das forças políticas organizadas, o poder público passou a atuar diretamente na pauta. Nesse momento, a ausência de abastecimento de água universal era percebida como um problema, a implantação de sistemas de água a serem geridos pelas comunidades se mostrava uma solução disponível para resolvê-lo e as condições políticas eram favoráveis à implementação dessa alternativa, caracterizando o acoplamento dos três fluxos que determinam a formação de agenda.

Dessa maneira, três grupos de agentes foram os principais influenciadores dos fluxos decisórios para o acoplamento: os moradores das áreas rurais, por meio do relato das dificuldades e da demanda contínua frente ao poder público local; os membros do SAAE, por meio da concepção e difusão da solução adotada; e o Executivo municipal, com a proposição de um projeto de governo voltado para a universalização do abastecimento de água. Nota-se que, sob a perspectiva do papel do saneamento na saúde da população, o início dos sistemas se deu por uma busca pela prevenção de doenças, e, com a atuação dos empreendedores, se expandiu para uma concepção de promoção da saúde a ser garantida a todos os moradores.

**Figura 3:**
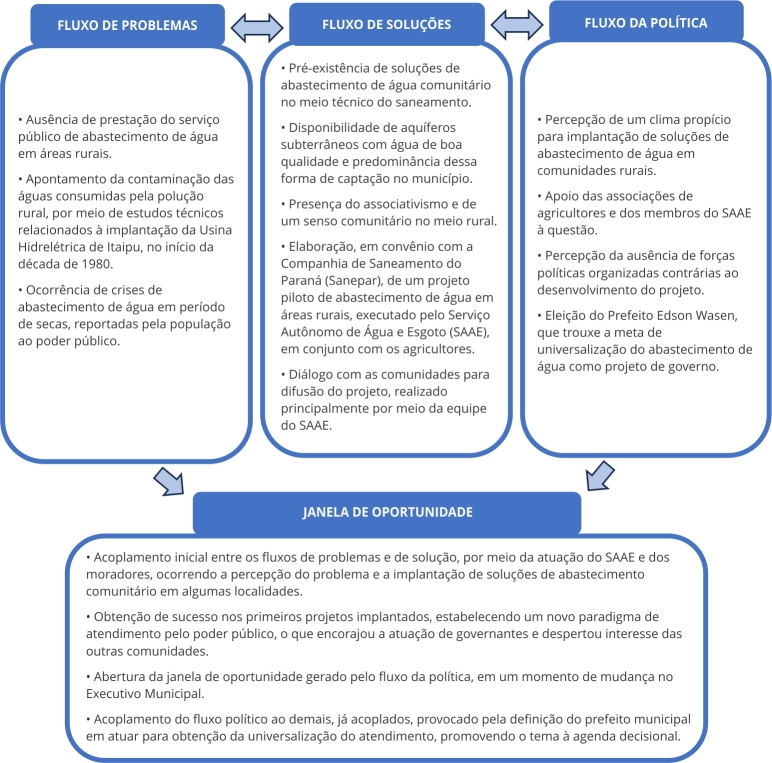
Fluxograma do processo de ascensão da universalização do abastecimento de água em comunidades rurais na agenda decisional de Marechal Cândido Rondon, Paraná, Brasil, de acordo com o Modelo de Fluxos Múltiplos.

A participação desses agentes é indicada na Figura 3, que apresenta uma síntese dos principais elementos identificados a partir do Modelo de Fluxos Múltiplos no processo de ascensão da universalização do abastecimento de água à agenda decisional de Marechal Cândido Rondon. A interrelação entre esses elementos, que é específica do contexto de Marechal Cândido Rondon, demonstra que as condicionantes locais influenciaram no desenvolvimento do processo em estudo.

## Conclusão

Este trabalho teve como objetivo analisar como o abastecimento de água universal em áreas rurais se tornou parte da agenda decisional em Marechal Cândido Rondon. A fundamentação teórica utilizada foi o Modelo de Fluxos Múltiplos de Kingdon. A aplicabilidade do modelo ao caso em estudo, já indicada pela literatura do setor, foi confirmada, tendo sido observado alinhamento entre os relatos dos entrevistados e os processos descritos por Kingdon.

A partir da análise de conteúdo qualitativa de entrevistas realizadas com informantes-chave, constatou-se que, devido à demanda dos moradores e a estudos de qualidade da água, o fluxo dos problemas foi responsável por atrair atenção do governo local ao tema do abastecimento de água na zona rural. Após o desenvolvimento de um projeto piloto de abastecimento comunitário, os agentes locais passaram a considerar a existência de uma alternativa viável e disponível, havendo o alinhamento com o fluxo de soluções. Essa solução foi propagada por membros do SAAE e se iniciou a expansão do atendimento a algumas comunidades rurais.

No fluxo da política, uma mudança no governo municipal propiciou a oportunidade para convergência dos três fluxos. O Prefeito Edson Wasen, ao assumir o cargo em 2001, definiu a universalização como uma meta de governo, o que, contando com um clima popular favorável, ascendeu o tema à agenda decisória. Já existindo um problema percebido como tal e uma solução disponível para ele, o poder público passou a atuar para efetivamente atender toda a população.

Também foi possível constatar que existiu uma diferença entre as decisões voltadas ao abastecimento de água e as decisões voltadas à universalização desse serviço. Enquanto o atendimento universal não fazia parte da agenda decisional, a atuação do poder público em Marechal Cândido Rondon, por mais que existente, não era suficientemente incentivada, tornando a expansão morosa e condicionada à evidenciação de problemas localizados. Tratar a universalização como um objetivo foi fundamental para sua obtenção.

Conforme demonstrado, condicionantes locais contribuíram para o êxito do caso de Marechal Cândido Rondon, o que pode limitar a replicabilidade, em outros contextos, das soluções ali adotadas. Apesar disso, ao apresentar a influência dos fluxos decisórios na ascensão do tema da universalização à agenda, esse estudo de caso evidencia para outros empreendedores a importância de identificar as particularidades locais e atuar para propiciar a convergência de fluxos. Além disso, a priorização da universalização, que se mostrou essencial, é um elemento replicável em diferentes realidades na busca pela promoção da saúde, sendo inclusive amparada pelas diretrizes nacionais do saneamento básico ^5^.
